# Large voltage-induced coercivity change in Pt/Co/CoO/amorphous TiO_*x*_ structure and heavy metal insertion effect

**DOI:** 10.1038/s41598-021-00960-w

**Published:** 2021-11-02

**Authors:** Tomohiro Nozaki, Shingo Tamaru, Makoto Konoto, Takayuki Nozaki, Hitoshi Kubota, Akio Fukushima, Shinji Yuasa

**Affiliations:** Research Center for Emerging Computing Technologies (RCECT), National Institute of Advanced Industrial Science and Technology (AIST), Tsukuba, Ibaraki 305-8568 Japan

**Keywords:** Electrical and electronic engineering, Magnetic properties and materials

## Abstract

There is urgent need for spintronics materials exhibiting a large voltage modulation effect to fulfill the great demand for high-speed, low-power-consumption information processing systems. Fcc-Co (111)-based systems are a promising option for research on the voltage effect, on account of their large perpendicular magnetic anisotropy (PMA) and high degree of freedom in structure. Aiming to observe a large voltage effect in a fcc-Co (111)-based system at room temperature, we investigated the voltage-induced coercivity (*H*_c_) change of perpendicularly magnetized Pt/heavy metal/Co/CoO/amorphous TiO_*x*_ structures. The thin CoO layer in the structure was the result of the surface oxidation of Co. We observed a large voltage-induced *H*_c_ change of 20.2 mT by applying 2 V (0.32 V/nm) to a sample without heavy metal insertion, and an *H*_c_ change of 15.4 mT by applying 1.8 V (0.29 V/nm) to an Ir-inserted sample. The relative thick Co thickness, Co surface oxidation, and large dielectric constant of TiO_*x*_ layer could be related to the large voltage-induced *H*_c_ change. Furthermore, we demonstrated the separate adjustment of *H*_c_ and a voltage-induced *H*_c_ change by utilizing both upper and lower interfaces of Co.

In response to the high demand for high-speed, low-power-consumption information processing systems, voltage-controlled spintronics devices such as a voltage-controlled magneto-resistive random access memory (MRAM)^1^ have attracted great attention. There is urgent need for materials exhibiting large voltage modulation effect for such applications. The voltage control of magnetic anisotropy (VCMA) effect^2,3^ and VCMA-induced precessional magnetization switching^4,5^, which are the core technologies of the voltage-controlled MRAM, have been extensively investigated, especially for bcc-Fe/MgO-based systems^6–9^, because of the high compatibility with MRAM. Regarding the purely-electronic VCMA effect, a VCMA coefficient of approximately 350 fJ/Vm has been realized for the epitaxial Cr/Fe/Ir/FeCo/MgO structure by precise control of the film structure using the molecular beam epitaxy (MBE) technique^10,11^. However, further enhancement of the VCMA coefficient is demanded. Fcc-Co (111)-based systems are a promising option, on account of its large perpendicular magnetic anisotropy (PMA) and high adjustability. Furthermore, it may achieve a large VCMA coefficient at room temperature. Thus far, a huge VCMA coefficient exceeding 1,000 fJ/Vm at low temperature has been demonstrated^12,13^. Even at room temperature, a large VCMA coefficient of 230 fJ/Vm was obtained^14^. These reports used polycrystalline samples prepared by sputtering. There is room for further improvement through the implementation of an epitaxial film or precise control of the film structure. Another advantage of Fcc-Co (111)-based systems is a large interface PMA, which arise at various interfaces^15^. In bcc-Fe-based systems, the Fe/MgO interface has been one of the few candidates to realize a large interface PMA^16,17^. Thus, Fe/MgO interfaces must meet the severe requirements of generating both a large PMA and VCMA. Contrastingly, in fcc-Co (111)-based systems, the PMA and VCMA can be adjusted separately by utilizing both the upper and lower interfaces of Co; that is, the Co (111)-based system has a high degree of freedom in structure. In addition, the large interface PMA enables investigation of the voltage effect in a wide range of Co thicknesses. These merits make the fcc-Co (111)-based systems attractive for VCMA research. One issue with investigating the voltage effect of the fcc-Co (111) system is the absence of a suitable dielectric layer material. While MgO (111) single dielectric layer^18,19^ and MgO (111)/amorphous HfO_*x*_ bilayer dielectric layers^20–25^ have been sometimes used for the voltage effect study, the large mismatch between MgO (111) and Co (111) and the natural polar plane of MgO make the realization of a high-quality interface difficult. Other crystal dielectric layers, such as Cr_2_O_3_^26^ and SrTiO_3_^27^, with less lattice mismatch, are difficult to grow on Co without degradation. An amorphous dielectric layer is another option, which can be used without concerning the lattice mismatch. Amorphous AlO_*x*_^28,29^ and HfO_*x*_^14,30^ dielectric layers have been used in the past. In this study, we focused on amorphous TiO_*x*_ as a dielectric layer material. We expected an enhancement in electron accumulation/depletion, due to a possibly large dielectric constant^31^.

In this study, we investigated the voltage-induced coercivity (*H*_c_) change in nominal Pt/Co/TiO_*x*_-based structures. First, we studied the influence of the Co thickness on the voltage-induced *H*_c_ change. Based on those results, we prepared samples with heavy metal (Ir, Ru, Ta, and W) insertion and investigated the PMA, voltage-induced *H*_c_ changes, and their annealing effect. A large *H*_c_ change was obtained for all the samples studied. Furthermore, we demonstrated separate adjustment of PMA and VCMA by utilizing both lower and upper interfaces of Co.

## Results and discussion

**Figure 1:**
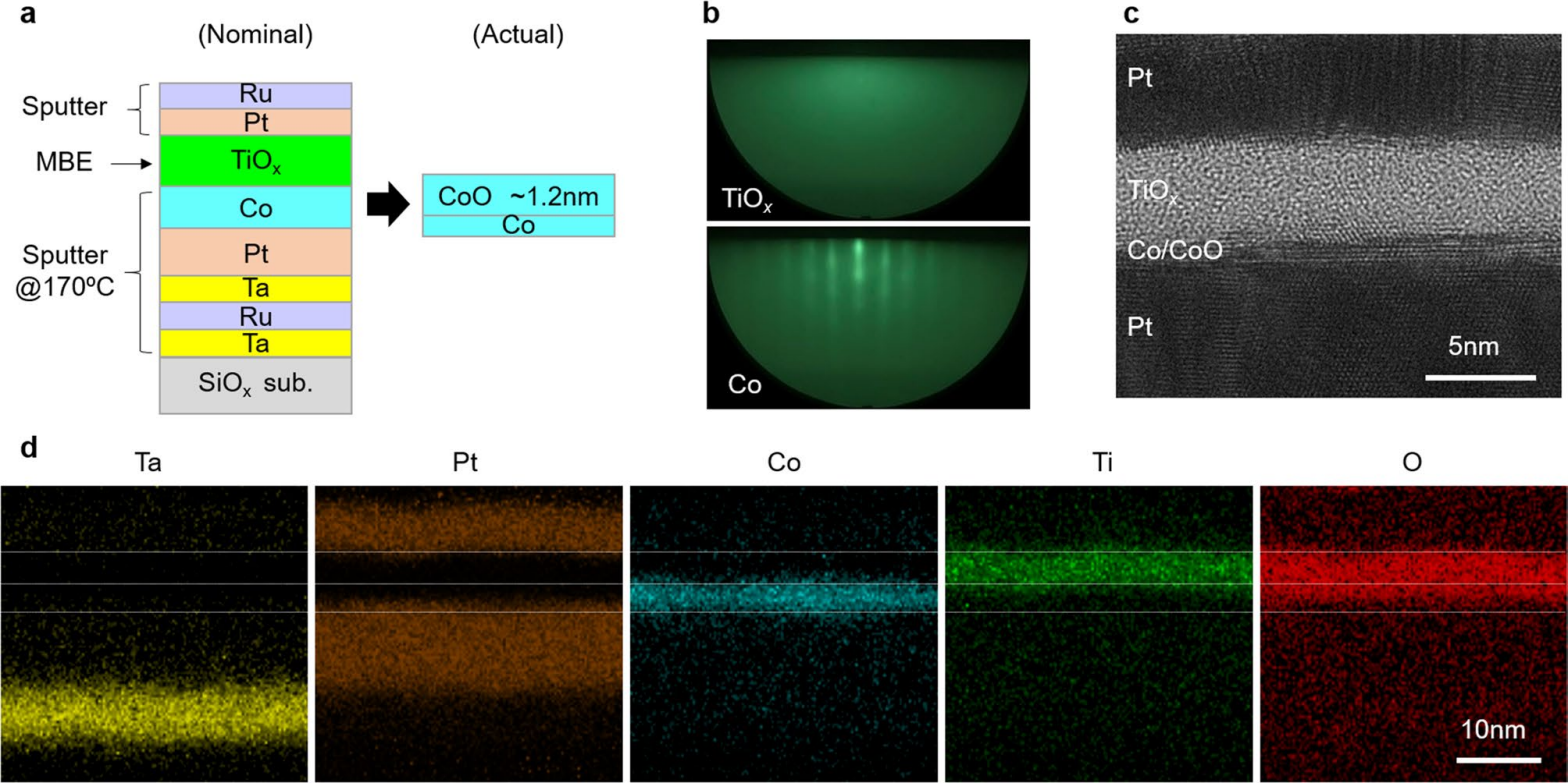
(**a**) Schematics of the nominal and estimated actual film structures, (**b**) RHEED patterns of Co (lower image) and TiO_*x*_ (upper image) layers, (**c**) cross sectional TEM image, and (**d**) EDX element maps (Ta, Pt, Co, Ti, and O) of nominal SiO_*x*_ sub./Ta (5 nm)/Ru (10 nm)/Ta (5 nm)/Pt (10 nm)/Co (1.2 nm)/TiO_*x*_ (3.8 nm)/Pt (5 nm)/Ru (2 nm) sample. The dotted white lines are visual guidelines indicating the approximate Ti and Co layer positions.

The actual film structure was evaluated from reflection high-energy electron diffraction (RHEED) and cross sectional transmission electron microscope (TEM) analysis. Figure 1(a) shows the schematics of the nominal structure used for the structural analysis. Figure 1(b) shows the RHEED patterns of the Co ferromagnetic layer (lower image) and TiO_*x*_ dielectric layer (upper image). A streak pattern corresponding to the (111) oriented texture was observed for the Co layer. On the other hand, no clear patterns were observed for the TiO_*x*_ layer, indicating that the TiO_*x*_ layer is amorphous. Figure 1(c) shows the cross-sectional TEM image of the Pt/Co/TiO_*x*_/Pt layers. The amorphous state of the TiO_*x*_ layer was confirmed by the TEM image. A thin crystal layer was observed at the Co/TiO_*x*_ interface, indicating the existence of a crystal CoO layer, which had formed during the O_2_ flow before TiO_*x*_ deposition. We separately confirmed the formation of a crystal CoO (111) texture on Co (111) by the O_2_ flow, from RHEED observations. Figure 1(d) shows the energy-dispersive X-ray spectroscopy (EDX) element map of Ta, Pt, Co, Ti, and O in the sample. The oxygen was distributed not only on the TiO_*x*_ layer but also on top of the Co layer, thus confirming the existence of the CoO layer at the interface. While oxygen was distributed on both Co and TiO_*x*_ layers, the interface between Ti and Co looked clear and intermixing was small. From the EDX element map, the thickness of the TiO_*x*_ and CoO layers were roughly estimated to be 3.8 nm and 1.2 nm, respectively. The estimated actual structure is also shown in Fig. 1(a). The element mapping also confirmed a finite intermixing at the Pt/Co interface, possibly occurs during sputter Co deposition.

**Figure 2:**
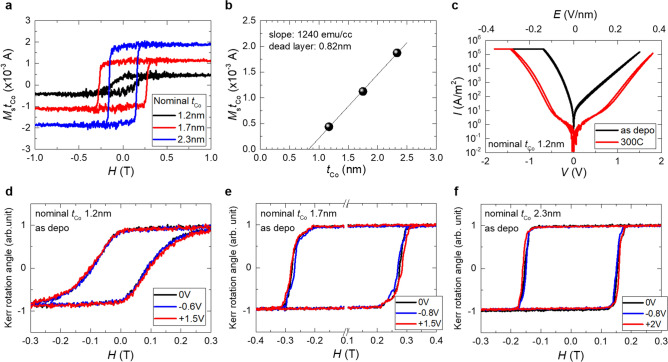
(**a**) Perpendicular magnetization curves of nominal SiO_*x*_ sub./Ta (5 nm)/Ru (10 nm)/Ta (5 nm)/Pt (10 nm)/Co (1.2, 1.7, and 2.3 nm)/TiO_*x*_ (3.8 or 5 nm)/Pt (5 nm)/Ru (2 nm) samples measured by VSM. (**b**) Co thickness dependence of the *M*_s_*t*_Co_ of the samples. (**c**) I–V characteristics of the as-deposited and 300 °C annealed sample with nominal Co thickness 1.2 nm. (**d**)–(**f**) Bias-voltage-dependence of the perpendicular magnetization curves of the samples with nominal Co thickness (**d**) 1.2 nm, (**e**) 1.7 nm, and (**f**) 2.3 nm, measured by MOKE.

Figure 2(a) shows the perpendicular magnetization curves of nominal SiO_*x*_ sub./Ta (5 nm)/Ru (10 nm)/Ta (5 nm)/Pt (10 nm)/Co (1.2, 1.7, or 2.3 nm)/TiO_*x*_ (3.8 or 5 nm)/Pt (5 nm)/Ru (2 nm) samples, measured using a vibrating sample magnetometer (VSM). All samples exhibited perpendicular magnetization with a large *H*_c_, possibly due to the interfacial PMA at the Pt/Co^15^ and Co/oxide^32–34^ interfaces. We also measured the in-plane magnetization curves of the samples. However, the saturation fields were larger than the maximum applied magnetic field (2 T) and we could not evaluate the magnetic anisotropy of these samples. The thickness dependence of the *M*_s_*t*_Co_, where *M*_s_ is saturation magnetization obtained from Fig. 2(a), are shown in Fig. 2(b). We estimated the magnetic dead layer thickness to be approximately 0.8 nm. The relatively large magnetic dead layer was mainly attributed to the Co oxidation at the Co/TiO_*x*_ interface. Considering the lattice parameters of Co and CoO (2.51 and 4.23 Å, respectively), the 1.2 nm CoO layer in Fig. 1 was formed by the oxidation of 0.7 nm-thick Co. The estimated oxidized Co layer thickness was approximately consistent with the magnetic dead layer obtained from the VSM measurements.

We microfabricated these samples and measured the bias-voltage-dependence of the perpendicular magnetization curve by magneto-optical Kerr effect (MOKE). A simple schematic of the microfabricated sample is shown in inset of Fig. 5(b). Figure 2(c) shows the I–V characteristics of the nominal Co 1.2-nm sample. The upper axis indicates the calculated applied electric field, assuming the dielectric layer thickness as the sum of CoO and TiO_*x*_ thicknesses (1.2 and 3.8 nm, respectively). A positive voltage indicates electron accumulation and a negative voltage indicates electron depletion at the Co/oxide interface. An asymmetric I–V characteristic, possibly due to the asymmetric CoO/TiO_*x*_ bilayer-dielectric layer, was observed for the as-deposited sample. By post annealing, the applicable voltage was increased and the asymmetry was weakened. Similar I–V characteristics were observed for other samples (presented in supplementary materials S1). Figures 2(d)–(f) show the bias-voltage-dependence of the perpendicular magnetization curves for as-deposited samples with nominal Co thickness of 1.2 nm (d), 1.7 nm (e), and 2.3 nm (f). Although the large *H*_c_ made it difficult to see the change, a distinguishable linear *H*_c_ change was observed by the voltage application. By positive (negative) voltage application, the *H*_c_ values of all the samples were increased (decreased). The sign of the voltage effect was consistent with that observed for surface oxidized Pt/Co/oxide systems^14,28^. A parabolic *H*_c_ change, induced by a Joule heating due to finite current by applying voltage^35,36^, was not clearly observed in this study. The respective *H*_c_ and Δ*H*_c_/Δ*V* values were 90 mT and 3.6 mT/V for the nominal Co 1.2-nm sample, 280 mT and 7.5 mT/V for the nominal Co 1.7-nm sample, and 150 mT and 3.0 mT/V for the nominal Co 2.3-nm sample. Thus far, the enhancement of VCMA or Δ*H*_c_/Δ*V* by Co surface oxidation has been reported several times^14,27,28^. We deduced that the Co surface oxidation also played an important role in the large Δ*H*_c_/ΔV observed in this study. The Δ*H*_c_/Δ*V* value changed non-monotonically against Co thickness; the decrease of Δ*H*_c_/Δ*V* for nominal Co 1.2-nm sample may be due to the degradation of the interfacial PMA, while the decrease of Δ*H*_c_/Δ*V* for nominal Co 2.3-nm sample may be due to the decrease of the interfacial PMA contribution (for details, please see supplementary information S2). As the results, the largest Δ*H*_c_/Δ*V* was obtained for the relatively thick Co sample (Co nominal 1.7-nm sample).

**Figure 3:**
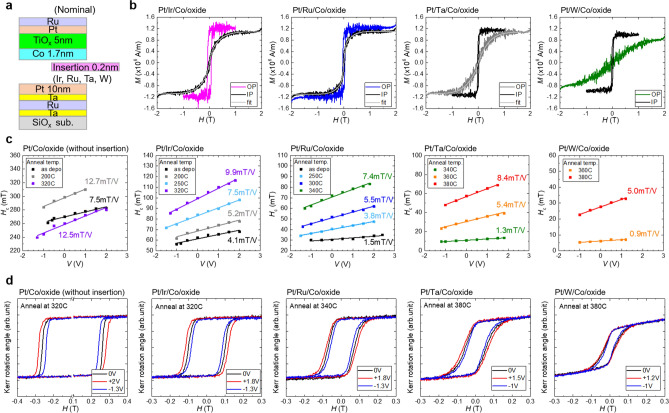
(**a**) Schematics of nominal sample structure (SiO_*x*_ sub./Ta (5 nm)/Ru (10 nm)/Ta (5 nm)/Pt (10 nm)/heavy metal insertion layer (0.2 nm)/Co (1.7 nm)/TiO_*x*_ (5 nm)/Pt (5 nm)/Ru (2 nm)). (**b**) Perpendicular (colored lines) and in-plane (black lines) magnetization curves of the heavy mental-inserted samples. The fitting curve used for evaluating magnetic anisotropy energy is also shown by light gray lines. (**c**) Bias-voltage-dependence of *H*_c_ of the samples at different annealing temperatures. (**d**) Bias-voltage-dependent perpendicular magnetization curves of the samples at a representative annealing temperature.

Next, we investigated the effect of heavy metal insertion on the *H*_c_ and Δ*H*_c_/Δ*V* using the nominal SiO_*x*_ sub./Ta (5 nm)/Ru (10 nm)/Ta (5 nm)/Pt (10 nm)/insertion layer (0.2 nm)/Co (1.7 nm)/TiO_*x*_ (5 nm)/Pt (5 nm)/Ru (2 nm) structure (where insertion layer = Ir, Ru, Ta, and W), as shown in Fig. 3(a). The insertion layer was introduced at the Pt/Co lower interface. We expected the insertion layer to effectively modulate the interface PMA at the Pt/Co lower interface, while maintaining a limited influence on the Δ*H*_c_/Δ*V* at the Co/oxide upper interface, as the bias-voltage was mainly applied to the Co/oxide upper interface. We also investigated the effect of post annealing, expecting to see the influence of the possible inter-diffusion of the inserted heavy metal layer on the Co ferromagnetic layer. Note that it was more straightforward to insert a heavy metal at the Co/oxide upper interface to observe the influence of the insertion layer on both PMA and VCMA, as the effectiveness has already been demonstrated for Fe/MgO based systems^10,11,37,38^. We also tested a heavy metal insertion at the Co/oxide upper interface, but found that the insertion layer at Co/oxide interface changed the optimum conditions for the Co surface oxidation. Further optimization of oxidation conditions would be required to clarify the effect.

Figure 3(b) shows the perpendicular (colored lines) and in-plane (black lines) magnetization curves of Ir-, Ru-, Ta-, and W-inserted samples before microfabrication, measured by VSM. Here, we assumed the actual Co thickness to be 0.9 nm. The 0.2 nm insertion layers effectively modulated (weakened) the PMA at the Pt/Co lower interface; weak perpendicular magnetization was obtained for Ir- and Ru-inserted samples, and in-plane magnetization was obtained for Ta- and W-inserted samples. The difference was attributed to the differences in the interfacial PMA at the insertion layer/Co interface, lattice strain, and crystallinity of Co. While the insertion layer could also affect the crystal orientation of Co, the effect of the 0.2 nm-thick insertion layers was negligible. We confirmed the (111) texture orientation of the Co layer for all the samples from the RHEED patterns (for details, please see supplementary information S3). As a result of the PMA modulation, the saturation field became measurable by the VSM. We evaluated the magnetic anisotropy energy of the samples from hard axis magnetization curves in Fig. 3(b). For an accurate evaluation, a fitting was performed using a function that reproduced the magnetization curve shape. The fitting curves are represented by the light gray lines in Fig. 3(b). The evaluated magnetic anisotropy energies of the Ir-, Ru-, and W-inserted samples were *K*^eff^ =  + 3.0 × 10^5^, + 1.6 × 10^5^, and − 3.8 × 10^5^ J/m^3^, respectively. We did not evaluate the magnetic anisotropy energy of the Ta-inserted sample, because its saturation field was larger than 2 T.

We microfabricated these samples and investigated the *H*_c_, Δ*H*_c_/Δ*V*, and the annealing effect. All the samples—except the W-inserted sample—exhibited similar I–V characteristics to those shown in Fig. 2(c). The W-inserted sample exhibited symmetric I–V characteristics, with lower resistivity (for details, please see Supplementary information S1). The applicable voltage was enhanced by post annealing for all the samples. Figure 3(c) summarizes the bias-voltage-dependence of the *H*_c_ of the samples (without insertion, and with Ir-, Ru-, Ta-, and W-insertion), at different annealing temperatures. The Δ*H*_c_/Δ*V* values obtained by the linear fitting are included the figure. Figure 3(d) displays the bias-voltage-dependent perpendicular magnetization curves of the samples at the representative annealing temperature. The sample without insertion is identical to that in Fig. 2(e). The Δ*H*_c_/Δ*V* of the sample without insertion increased by post annealing. The maximum value is approximately 12.7 mT/V, obtained by 200 °C annealing. At the optimum annealing temperature (Fig. 3d), a much larger voltage-induced *H*_c_ change than that of the as-deposited sample (Fig. 2d) was observed, reflecting the increase in both the Δ*H*_c_/Δ*V* and applicable voltage. The maximum *H*_c_ change observed was 20.2 mT by the application of 2 V (0.32 V/nm). The Ir- and Ru-inserted samples also exhibited a linear *H*_c_ change by bias-voltage application, even in the as-deposited state. The Δ*H*_c_/Δ*V* was 4.1 mT/V and 1.5 mT/V for Ir- and Ru-inserted samples, respectively. Here, we evaluated the VCMA coefficient of the as-deposited samples by VCMA ~ *K*^eff^*t*_Co_^eff^(Δ*H*_c_/Δ*V*)*t*_ox_/*H*_c_, assuming a linear *H*_c_ change corresponding to the change in interfacial PMA. Assuming *t*_Co_^eff^ = 0.9 nm and *t*_ox_ = *t*_CoO_ + *t*_TiOx_ = 6.2 nm, we obtained acceptable VCMA coefficients of approximately 107 fJ/Vm and 44 fJ/Vm, for as-deposited Ir- and Ru-inserted samples, respectively. By post annealing, both *H*_c_ and Δ*H*_c_/Δ*V* of the Ir- and Ru-inserted samples were largely enhanced; thus, the VCMA coefficient could also be largely enhanced. At the optimum annealing temperature, a much smaller *H*_c_ and close Δ*H*_c_/Δ*V* were observed compared with the sample without insertion (see Fig. 3d). The maximum voltage-induced *H*_c_ change observed for Ir-inserted samples was 15.4 mT by the application of 1.8 V (0.29 V/nm). While the Ta- and W-inserted samples exhibited in-plane magnetization in the as-deposited state, both perpendicular *H*_c_ and its linear voltage modulation (Δ*H*_c_/Δ*V*) were observed after high temperature annealing. However, the Δ*H*_c_/Δ*V* value of Ta- and W-inserted samples were not quantitative, because these magnetization curves had both perpendicular and in-plane components, as shown in Fig. 3(d).

**Figure 4:**
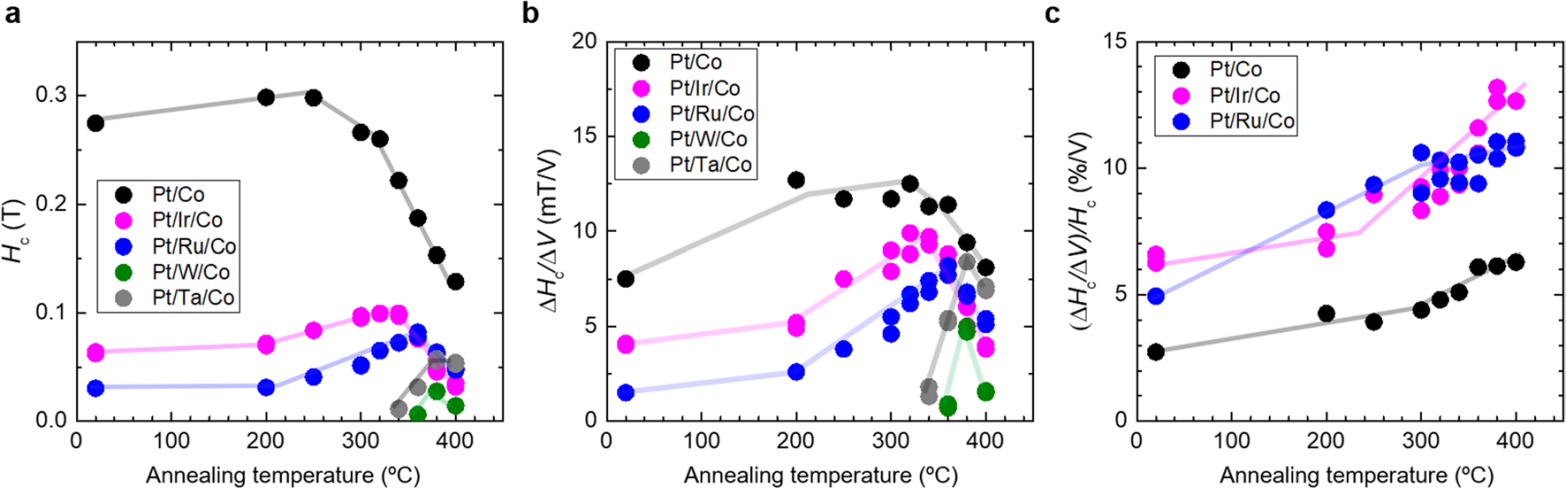
Annealing temperature-dependence of (**a**) *H*_c_, (**b**) Δ*H*_c_/Δ*V*, and (**c**) (Δ*H*_c_/Δ*V*)/*H*_c_ of the samples without and with heavy metal insertion.

Figure 4 (a) and (b) shows the annealing temperature dependence of *H*_c_ and Δ*H*_c_/Δ*V* of the samples, respectively. With increasing post annealing temperature, initially both *H*_c_ and Δ*H*_c_/Δ*V* gradually increased, then both decreased above a certain temperature. Interestingly, with increasing annealing temperature, the Δ*H*_c_/Δ*V* of samples with heavy metal insertion approached that of the samples without insertion. As the Δ*H*_c_/Δ*V* is governed by the Co/oxide upper interface, this indicates that a similar Co/oxide upper interface was realized in both samples (without and with insertion) after post annealing. However, the *H*_c_ values of samples with heavy metal insertion were much smaller than those of the samples without insertion, even after post annealing. Hence, we demonstrated the effectiveness of the heavy metal insertion for adjusting PMA at the Pt/heavy metal/Co lower interface, while maintaining the voltage-induced *H*_c_ change at the Co/oxide upper interface. This indicates the realization of independent adjustment of PMA and VCMA in Pt/Co/oxide systems. Figure 4(c) shows the annealing temperature dependence of (Δ*H*_c_/Δ*V*)/*H*_c_—a percentage of *H*_c_ modulation by application of 1 V—of samples without insertion and with Ir and Ru insertion. Owing to the abovementioned independent adjustment, the (Δ*H*_c_/Δ*V*)/*H*_c_ value greatly improved in the entire annealing temperature range due to the Ir and Ru insertion. On the other hand, we did not observe an evident increase in Δ*H*_c_/Δ*V*, which could be attributed to possible inter-diffusion of the inserted heavy metal, in this study.

**Figure 5:**
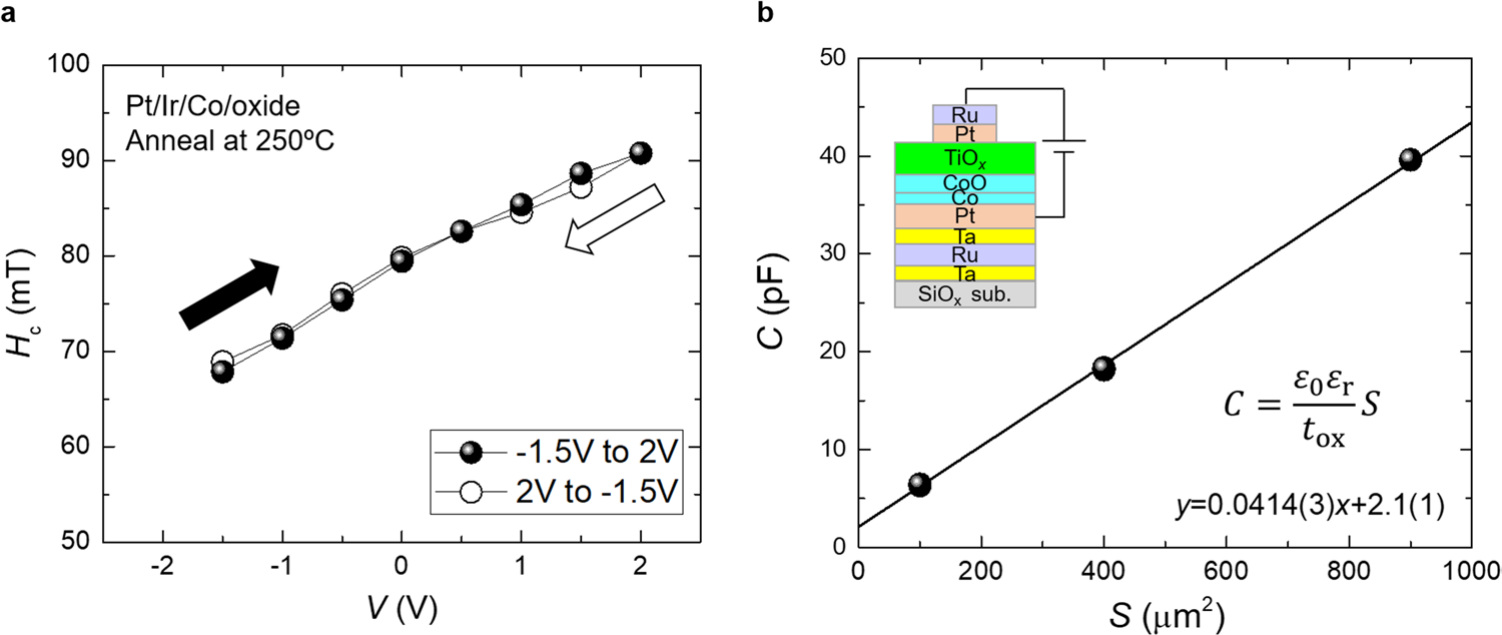
(**a**) Bias-voltage-dependence of *H*_c_ of Ir-inserted sample (post annealed at 250 °C). The bias-voltage was applied in order: from − 1.5 V to 2.0 V (ascending branch, black closed circles) and from 2.0 V to − 1.5 V (descending branch, black open circles). (**b**) Device size (*S*) dependence of device capacitance (*C*) of nominal SiO_*x*_ sub./Ta (5 nm)/Ru (10 nm)/Ta (5 nm)/Pt (10 nm)/Co (1.7 nm)/TiO_*x*_ (5 nm)/Pt (5 nm)/Ru (2 nm)) sample. The simple schematics of the device is shown in the inset.

In addition to the independent adjustment, the large voltage-induced *H*_c_ changes were also notable results. We observed a large voltage-induced *H*_c_ change of 20.2 mT for the sample without insertion by the application of 2 V (0.32 V/nm), and 15.4 mT for the sample with Ir-insertion by applying 1.8 V (0.29 V/nm). These results were more than an order of magnitude larger than those reported in Pt/Co/oxide systems at room temperature when an electric field of up to 0.38 V/nm was applied^13,20,21,27^. A *H*_c_ change larger than 10 mT by voltage applications has been reported for Pt/Co/oxide systems when measured at low temperatures^28,30^, or when the apparent magneto-ionic (redox) effect was observed^14,39,40^. In the latter case, a non-volatile *H*_c_ change with a very slow time scale (more than several minutes) has been observed on applying bias-voltage. The *H*_c_ change in this study was faster than the MOKE measurement time (< 1 min), and the possibility of the magneto-ionic effect was low. Figure 5(a) shows the bias-voltage-dependence of the *H*_c_ of the Ir-inserted sample (post annealed at 250 °C). The bias-voltage was increased from − 1.5 V to 2.0 V (ascending branch, black closed circles), and then decreased from 2.0 V to − 1.5 V (descending branch, black open circles). No apparent bias-voltage hysteresis of *H*_c_ was observed, thus denying an evident magneto-ionic effect.

Finally, we evaluated the dielectric constant of the CoO/TiO_*x*_ dielectric layers. Figure 5(b) shows the device size (*S*) dependence on the capacitance (*C*) of the nominal SiO_*x*_ sub./Ta (5 nm)/Ru (10 nm)/Ta (5 nm)/Pt (10 nm)/Co (1.7 nm)/TiO_*x*_ (5 nm)/Pt (5 nm)/Ru (2 nm)) sample. 1.2-nm CoO and 5-nm TiO_*x*_ bilayer dielectrics were expected to exist. When the device size was small, non-negligible capacitance components, other than the dielectric layers, were clearly superimposed. We eliminated the other components by measuring the device size dependence (100, 400, and 900 μm^2^). The capacitance varied linearly against the device size, with a slope of *C*/*S* = 0.0414 F/m^2^. Assuming *t*_ox_ = 6.2 nm, the averaged relative dielectric constant *ε*_r_ (= (*t*_ox_*C*)/(*ε*_0_*S*)) was estimated to be 29. The *ε*_r_ is approximately 3 times larger than that of MgO (*ε*_r_ = 9.8). The large *ε*_r_ may enhance the voltage effect of the samples in this study. Note that the *ε*_r_ of TiO_*x*_ itself should be larger, because the thin CoO layer decreases the averaged *ε*_r_ (more details, see supplementary information S4).

We speculated that, in addition to the large dielectric constant, the relative thick Co thickness (~ 0.9 nm after oxidation) and surface oxidation of Co had important roles in the observation of the large voltage-induced *H*_c_ change. Regarding the former, we observed a large Co thickness variation with the *H*_c_ change, as shown in Fig. 2(d)–(f). As discussed in supplementary information S2, when the Co thickness is too thin, the *H*_c_ and Δ*H*_c_/Δ*V* degrade possibly due to the degradation of the interfacial PMA. In association, a large electric field effect on the magnetic domain wall velocity through the modulation of the pining potential has recently been reported for relative thick Co thickness sample^24,25^. These results indicate the importance of the optimization of the Co thickness. Regarding the latter, the mechanism of the enhancement of VCMA or voltage-induced *H*_c_ change due to Co surface oxidation has not yet been clarified, although there have been several reports of experimental demonstration^14,27,28^. We confirmed that CoO (111) texture was formed on Co (111) after the surface oxidation. Since a lattice strain due to the lattice mismatch should exist at the Co/CoO interface, the lattice strain at the interface could be related to the large *H*_c_ change. A detailed analysis using an epitaxial thin film, combined with first principle calculations, could shed more light on the elucidation of the mechanism.

## Conclusions

In this study, we investigated the voltage-induced *H*_c_ change of perpendicularly magnetized Pt/heavy metal/Co/CoO/amorphous TiO_*x*_ structures. The insulating TiO_*x*_ dielectric layer was obtained by oxygen radical-assisted MBE, with a very slow metal Ti evaporation rate. A thin CoO layer was formed by the surface oxidation of Co. We demonstrated a voltage-induced *H*_c_ change larger by about an order of magnitude than those in previous reports: 20.2 mT and 15.4 mT by application of 1.8–2 V (0.29–0.32 V/nm) for samples without heavy metal insertion and with Ir-insertion, respectively. The relative thick Co thickness (about 0.9 nm), Co surface oxidation, and large dielectric constant (*ε*_r_ ~ 29) of CoO/TiO_*x*_ dielectric layers could be related to the large voltage-induced *H*_c_ change. Furthermore, we demonstrated the separate adjustment of *H*_c_ and voltage-induced *H*_c_ change by utilizing both the lower and upper interfaces of Co. These results show the potential of a large VCMA effect at room temperature and the good controllability of the PMA and VCMA in the fcc-Co (111)-based system. Based on our results, further developments are expected for the voltage effect of the system.

## Methods

A nominal structure of SiO_*x*_ sub./Ta (5 nm)/Ru (10 nm)/Ta (5 nm)/Pt (10 nm)/insertion layer/Co (*t*_Co_ nm)/TiO_*x*_ (*t*_TiOx_ nm)/Pt (5 nm)/Ru (2 nm) was prepared using a combination of MBE and sputtering techniques. Before deposition, the SiO_*x*_ substrate was cleaned by in situ annealing at 300 °C. The Ta/Ru/Ta/Pt bottom electrodes, insertion layers, and Co ferromagnetic layer were deposited by sputtering at 170 °C. The TiO_*x*_ dielectric layer was prepared using an oxygen radical-assisted MBE technique. Ti metal was e-beam evaporated in an O_2_ atmosphere with a radio-frequency (RF) radical source at room temperature. The oxygen flow rate and RF power during TiO_*x*_ deposition were fixed at 0.5 sccm (~ 1.0 × 10^–3^ Pa) and 200 W, respectively. Our preliminary experiments showed that sufficiently strong oxidation is required to obtain an insulating TiO_*x*_ dielectric layer. In this study, we obtained the insulating TiO_*x*_ layer by utilizing the RF oxygen radical source while maintaining a very low Ti evaporation rate (< 0.02 Å/s). Before the deposition of the TiO_*x*_ layer, O_2_ flow was maintained with the main shutter closed for a while to establish stable oxygen flow rate, oxygen plasma, and deposition rate. This O_2_ flow causes surface oxidation of Co. We estimated approximately 1.2 nm-thick CoO layer was formed at the interface (see Fig. 1). Since the CoO layer is passivated, the CoO thickness is constant regardless of the Co thickness. Pt/Ru cap layers were deposited by sputtering at room temperature. The TiO_*x*_ thickness of the sample for structural analysis (Co nominal 1.2 nm sample) was estimated to be approximately 3.8 nm from cross sectional TEM analysis. The TiO_*x*_ thickness of the other samples was designed to be approximately 5.0 nm, which was controlled by monitoring deposition time and Ti evaporation rate using a quartz crystal microbalance, calibrated by the TEM analysis results. Because of the difficulty in estimating the exact thickness of the dielectric CoO and TiO_*x*_ layers, discussions in this study basically use voltage (V) rather than electric field (V/nm). The crystal orientation of the thin film was confirmed using RHEED in situ. Cross sectional TEM and EDX element mapping were performed to evaluate the actual film structures. The Pt/Ru cap layer of the film samples was micro-fabricated into a pillar of about 8 × 10 μm^2^. After backfilling with SiO_2_, the Cr/Au top electrode was formed. The micro-fabrication process is identical to that in our previous report^26^. After the microfabrication, the samples were post annealed at up to 400 °C in a vacuum furnace. Since a rapid degradation of the device resistance was observed after post annealing at high temperature (*T*_anneal_ > 360 °C), the temperature of post annealing was limited up to 400 °C (please see also supplementary information S1). The voltage-induced *H*_c_ change of each micro-fabricated sample was evaluated from out-of-plane magnetization curves measured by MOKE at room temperature. In the voltage effect measurements, unless otherwise stated, positive and negative voltages were applied alternately to eliminate the possible time-dependent change in *H*_c_. The magnetic anisotropy of the film sample was evaluated using VSM measured at room temperature. The capacitance of the microfabricated device was measured at room temperature by applying a triangular voltage waveform and capturing the current flowing into the device, which is a time-derivative of the voltage, and thus, becomes a square pulse with an amplitude proportional to the capacitance. This unique technique provided a highly precise capacitance value of a grounded device without requiring system calibration. The details of this technique will be presented in a separate paper.

## Supplementary Information

Supplementary Information.
