# Supplementary material for: Large voltage-induced coercivity change in Pt/Co/CoO/amorphous TiOx structure and heavy metal insertion effect
Source: Sci Rep. 2021 Nov 2;11:21448. doi: 10.1038/s41598-021-00960-w (PMC8564507; doi:10.1038/s41598-021-00960-w)
Supplement: Supplementary file 1 — Supplementary Information. [file 41598_2021_960_MOESM1_ESM.pdf]

Supplementary material

Large voltage-induced coercivity change in Pt/Co/CoO/amorphous TiO_x structure and heavy metal insertion effect

Tomohiro Nozaki[1*], Shingo Tamaru[1], Makoto Konoto[1], Takayuki Nozaki[1], Hitoshi Kubota[1], Akio Fukushima[1], and Shinji Yuasa[1]

[1]Research Center for Emerging Computing Technologies (RCECT), National Institute of Advanced Industrial Science and Technology (AIST), Tsukuba, Ibaraki 305-8568, Japan

*email:nozaki.tomohiro@aist.go.jp

S1 I-V characteristics of heavy metal inserted samples

Figure S1 (a)-(f) shows I-V characteristics of the sample without and with heavy metal insertion. Except W inserted sample, asymmetric I-V characteristics was observed, while the degree differ for each sample. By post annealing, the asymmetry was weakened and the applicable voltage was increased. In contrast, the W inserted sample exhibits a symmetric I-V characteristics with lower resistivity. Fig. S1 (g) summarize the post anneal temperature dependence of the resistivity @ 1 V of samples without and with heavy metal insertion (nominal t_{Co} = 1.7 nm samples). The resistivity increased by post annealing up to ~350 ºC. After post annealing at higher temperature, the resistivity degraded.

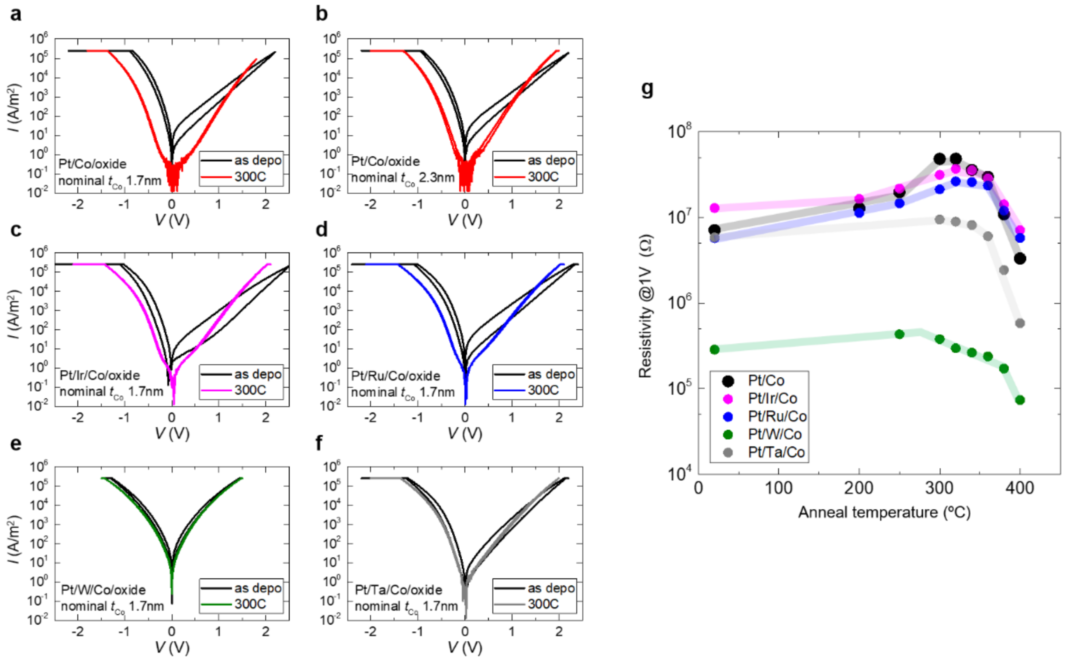

Fig. S1 (a)-(f) I-V characteristics of the sample without and with heavy metal insertion. The black lines and colored lines corresponding to those of as-deposited and post annealed samples. (g) Annealing temperature dependence of the resistivity @ 1 V of samples without and with heavy metal insertion. The nominal film structures are SiO_x sub./Ta (5nm)/Ru (10 nm)/Ta (5 nm)/Pt (10 nm)/heavy metal insertion layer (0.2 nm)/Co (1.7 or 2.3 nm)/TiO_x (5 nm)/Pt (5 nm).

S2 Co thickness dependence of H_c of Pt/Co/oxide sample

Figure S2 shows t_{Co} dependence of the product of H_c and t_{Co}^{eff} (= t_{Co} – t_{Co}^{dead}) of nominal SiO_x sub./Ta (5 nm)/Ru (10 nm)/Ta (5 nm)/Pt (10 nm)/Co (t_{Co} nm)/TiO_x (5 nm)/Pt (5 nm) sample measured by MOKE. Note that the sample is slightly different from samples in the main text; the Co ferromagnetic layer was deposited by thermal evaporation for the present sample, while the Co layer was deposited by sputter for samples in the main text. Thus, the magnetic properties of the present sample are slightly different from those in the main text. The magnetic dead layer thickness, t_{Co}^{dead}, was evaluated from Kerr rotation angle vs t_{Co} plot. If we assume the H_c is proportional to K^{eff} in this t_{Co} region, the plot in Fig. S2 correspond to $K^{eff}t_{Co}^{eff}$ vs t_{Co} plot. For thick Co region (t_{Co} > 1.7 nm), the $H_c t_{Co}^{eff}$ linearly increase with decreasing t_{Co}, indicating the constant contribution of the interfacial PMA in this region. On the other hand, for thinner Co region (t_{Co} < 1.7 nm), the $H_c t_{Co}^{eff}$ decrease with decreasing t_{Co}, indicating the degradation of the interfacial PMA. These trends are typical for $K^{eff}t_{FM}^{eff}$ vs t_{FM} plots in systems with a large interfacial PMA. With these trends in mind, for thick Co region, the H_c and $\Delta H_c/\Delta V$ will decrease due to decrease of the interfacial PMA contribution. For thin Co region, the H_c and $\Delta H_c/\Delta V$ will decrease due to the degradation of the interfacial PMA. Thus large H_c and $\Delta H_c/\Delta V$ will be obtained for relative thick Co thickness.

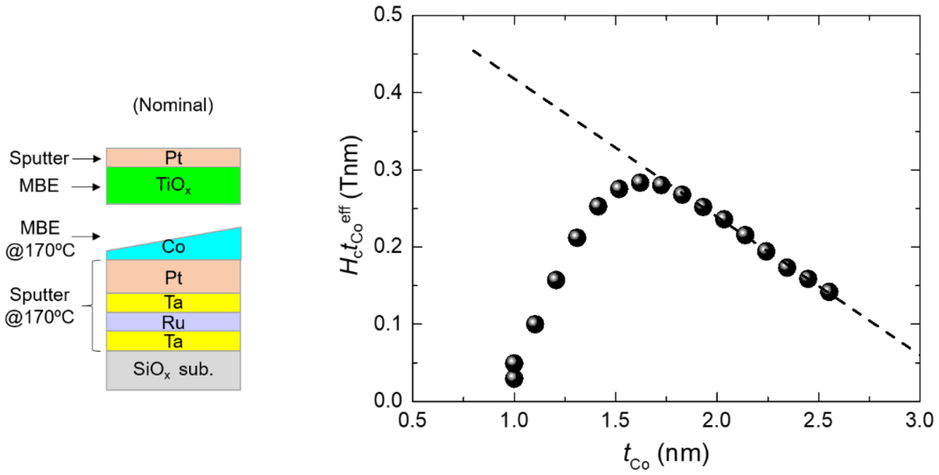

Fig. S2 Co thickness dependence of the product of H_c and t_{Co}^{eff} of nominal SiO_x sub./Ta (5 nm)/Ru (10 nm)/Ta (5 nm)/Pt (10 nm)/Co (t_{Co} nm)/TiO_x (5 nm)/Pt (5 nm) sample measured by MOKE. The schematics of the nominal structure is also drawn.

S3 RHEED patterns of Co in heavy metal inserted samples

Figure S3 shows the RHEED patterns of Co surface (before TiO_x deposition) in heavy metal 0.2 or 1 nm inserted samples. The RHEED patterns of Co in heavy metal 0.2 nm samples looks identical to that in Fig. 1 (b), confirming (111) texture orientation of Co for all samples. On the other hand, an obvious degrade of the streaky RHEED patterns was observed for Ta 1 nm inserted samples. These results indicate that the effect of 0.2 nm-thick insertion layers to the crystal orientation of Co were small, regardless the preferable crystal structure of the inserted heavy metals.

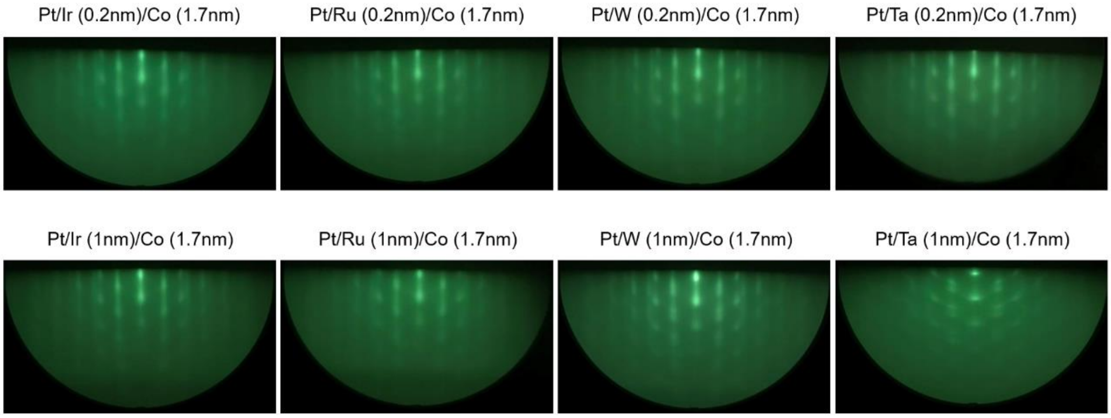

Fig. S3 RHEED patterns of Co surface in heavy metal 0.2 nm inserted structures (upper images) and 1 nm inserted structures (lower images). The nominal film structures are SiO_x sub./Ta (5nm)/Ru (10 nm)/Ta (5 nm)/Pt (10 nm)/heavy metal insertion layer (0.2 or 1 nm)/Co (1.7 nm).

S4 dielectric constant of bilayer dielectric layers

An averaged dielectric constant of bilayer dielectric layers are represented by the following equation;

$$\text{averaged } \varepsilon_r = \left(\frac{1}{\varepsilon_{r1}}\frac{d_1}{d_1+d_2} + \frac{1}{\varepsilon_{r2}}\frac{d_2}{d_1+d_2}\right)^{-1} \quad \text{(S1)}$$

Here, ε_{r1}(ε_{r2}) and d_1(d_2) represent relative dielectric constant and thickness of dielectric layer 1 (2), respectively. Fig. S4 shows d_2 dependence of averaged ε_r of bilayer dielectric layers, assuming ε_{r1} = 50, ε_{r2} = 10, and d_1 = 5 nm. We suppose dielectric layer 1 and 2 as a high-k TiO_x barrier and interfacial CoO layer, respectively. Fig. 3 shows a large influence of thin interfacial CoO layer on the average ε_r. Assuming 1.2-nm CoO layer at the interface, the ε_r of TiO_x layer is estimated to be ~50.

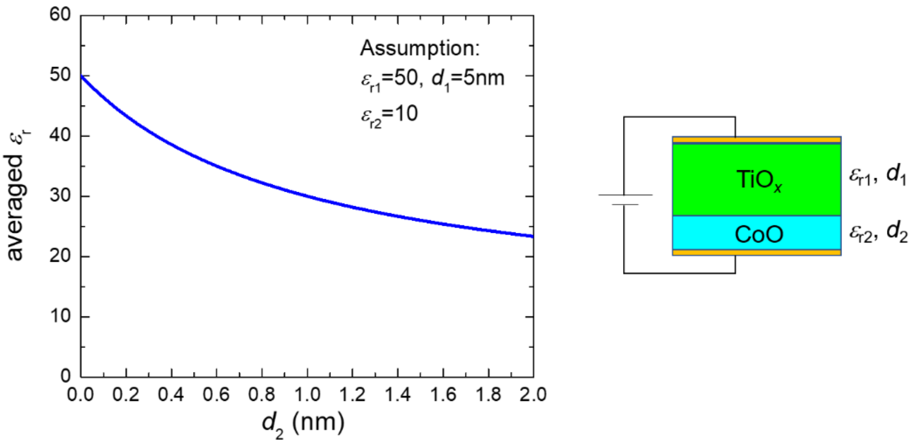

Fig. S4 d_2 dependence of averaged ε_r of bilayer dielectrics calculated by eq. (S1), assuming ε_{r1} = 50, ε_{r2} = 10, and d_1 = 5 nm.
